# Elevated AEG-1 expression in macrophages promotes hypopharyngeal cancer invasion through the STAT3-MMP-9 signaling pathway

**DOI:** 10.18632/oncotarget.12886

**Published:** 2016-10-25

**Authors:** Xiuxiu Liu, Zhenghua Lv, Jidong Zou, Xianfang Liu, Juke Ma, Chengtao Sun, Na Sa, Wei Xu

**Affiliations:** ^1^ Department of Otorhinolaryngology Head and Neck Surgery, Shandong Provincial Hospital Affiliated to Shandong University, Jinan, Shandong, China; ^2^ Shandong Provincial Key Laboratory of Otology, Jinan, Shandong, China

**Keywords:** macrophages, cancer invasion, AEG-1, MMP-9, hypopharyngeal cancer

## Abstract

Macrophages play a critical role in tumor invasion and metastasis, which remain major causes of mortality in patients with hypopharyngeal cancer. Here we investigate the effect of an oncogene, AEG-1 expressed in macrophages on the invasion of hypopharyngeal cancer cells. AEG-1 is more highly expressed in macrophages of human hypopharyngeal cancer samples compared with adjacent non-tumor controls. Using matrigel invasion assay system, THP-1-derived macrophages with forced AEG-1 overexpression enhance FaDu cell invasion whereas macrophages with AEG-1 silence inhibit. Matrix metalloproteinase 9 (MMP-9), which is important in tumor invasion and metastasis through degrading extracellular matrix, is up-reulated by AEG-1 partly through NF-κB p65 in macrophages. Intriguingly, macrophage AEG-1 also induces MMP-9 up-regulated expression in FaDu cells. Furthermore, macrophage AEG-1 activates signal transducer and activator of transcription 3 (STAT3) in FaDu cells, which is responsible for macrophage AEG-1-induced an increase in MMP-9 expression and invasion of FaDu cells. This is the first to demonstrate that macrophage AEG-1 promotes tumor invasion through up-regulation of MMP-9 in both macrophages and cancer cells. Thus, the results provide evidences that macrophage AEG-1 contributes to promotion of tumor invasion, and represents as a potential target in hypopharyngeal cancer therapy.

## INTRODUCTION

Tumor metastasis remains a major cause of cancer-related mortality in hypopharyngeal cancer [1, 2]. Despite great improvements in the diagnosis and therapy, the five-year survival rate is still poor. Thus, it is necessary and urgent to explore the underlying mechanism driving metastasis in hypopharyngeal cancer. Since metastasis is distant invasion [3], it is critical to understand the mechanisms of invasion.

Tumor associated macrophages (TAM), the abundant immune cells recruited to the tumor microenvironment, are recognized as key players in tumor invasion and metastasis [4]. Clinical evidences have shown that a high increase in infiltration of TAM frequently correlates with lower survival rates, and these observations indicate that TAM may promote tumor progression [5, 6]. In head and neck squamous cell carcinoma (HNSCC), the presence of macrophages in primary tumors is found to be an independent predictor of lymph node metastasis [7]. Activated macrophages are essential for tumor invasion via production of several matrix-metalloproteases (MMPs) such as MMP-9, which could destroy basement membrane and degrade the extracellular matrix (ECM), and thereby create a microenvironment conductive to tumor progression [8]. TAM also produce some factors, such as interleukin-6 (IL-6), tumour necrosis factor-α (TNF-α), which may activate some important signaling pathways in cancer cells and stimulate cancer metastasis [9, 10]. Given the importance of TAM in tumor development, there are more and more therapeutic strategies against cancer which are designed to target key molecules in the macrophages [11–13]. For example, a colony stimulating factor-1 receptor (CSF-1R) inhibitor is used to target TAM and thus results in a robust decrease in tumor volume along with improved survival in preclinical trials of glioma [11]. In recent years, a growing number of molecules, usually detected in tumor cells, were also found to be expressed in macrophages and critical for tumor pathogenesis [12–14]. Genetic studies in mice reveal that loss of epidermal growth factor receptor (EGFR) in liver macrophages impaired hepatocellular carcinoma (HCC) formation whereas loss of EGFR in hepatocytes unexpectedly increased proliferation and thus led to HCC development [12]. This explains why patients with advanced stage HCC do probably not benefit from EGFR-targeted therapies. Receptor for advanced glycation endproducts (RAGE) ablation in macrophages results in a significant prolongation in survival of glioma-bearing mice owing to a reduction in TAM-associated inflammation and angiogenesis [13]. Therefore, further studies are expected to look for the key molecules in macrophages to identify their effects on tumor cell invasion, and thereby provide potential therapeutic targets against cancer.

Astrocyte elevated gene-1 (AEG-1), also known as Metadherin (MTDH) and LYRIC, is an oncogene that is frequently overexpressed in various human cancers [1, 15]. Altered expression of AEG-1 is associated with worse prognosis in patients with various malignant tumors [15]. AEG-1 overexpression promoted tumorigenesis by its ability to stimulate angiogenesis and tumor cell invasion, while AEG-1 absence inhibited invasion and *in vitro* metastasis properties of tumor cells [14, 16, 17]. Strong evidence shows that AEG-1 markedly increased interaction with the p65 subunit of NF-κB that induced the expression of NF-κB downstream genes in HeLa cells [18] and inhibition of NF-κB activation in macrophages abrogated initiation and progression of HCC in AEG-1 knockout mice [14]. So far, the majority of studies regarding AEG-1 signaling in tumors have focused on cancer cell types. However, the functional consequences of AEG-1 in non-tumor cells, like macrophages, on tumor cell invasion and the underlying mechanisms have not been investigated in details.

In this study, we found higher expression level of AEG-1 in macrophages of hypopharyngeal cancer compared to adjacent non-tumor tissue. It is demonstrated that forced overexpression of AEG-1 in macrophage promoted the invasion of hypopharyngeal cancer cells using a matrigel invasion assay system. The up-regulation of MMP-9 by AEG-1 in macrophages was mediated through NF-κB p65. Furthermore, macrophage AEG-1 activated STAT3-MMP-9 pathway in FaDu cells, ultimately promoting cancer cell invasion. This study is the first to demonstrate that macrophage AEG-1 not only induced MMP-9 expression in macrophages but also up-regulated MMP-9 expression in cancer cells. Our findings here provided new insights into the functional contributions of macrophage AEG-1 on the invasion of hypopharyngeal cancer cells, thus laying a foundation for developing drugs targeting AEG-1 in macrophages in the future.

## RESULTS

### The expression of AEG-1 was significantly higher in macrophages and associated with elevated expression levels of MMP-9 in hypopharyngeal cancer

**Figure 1:**
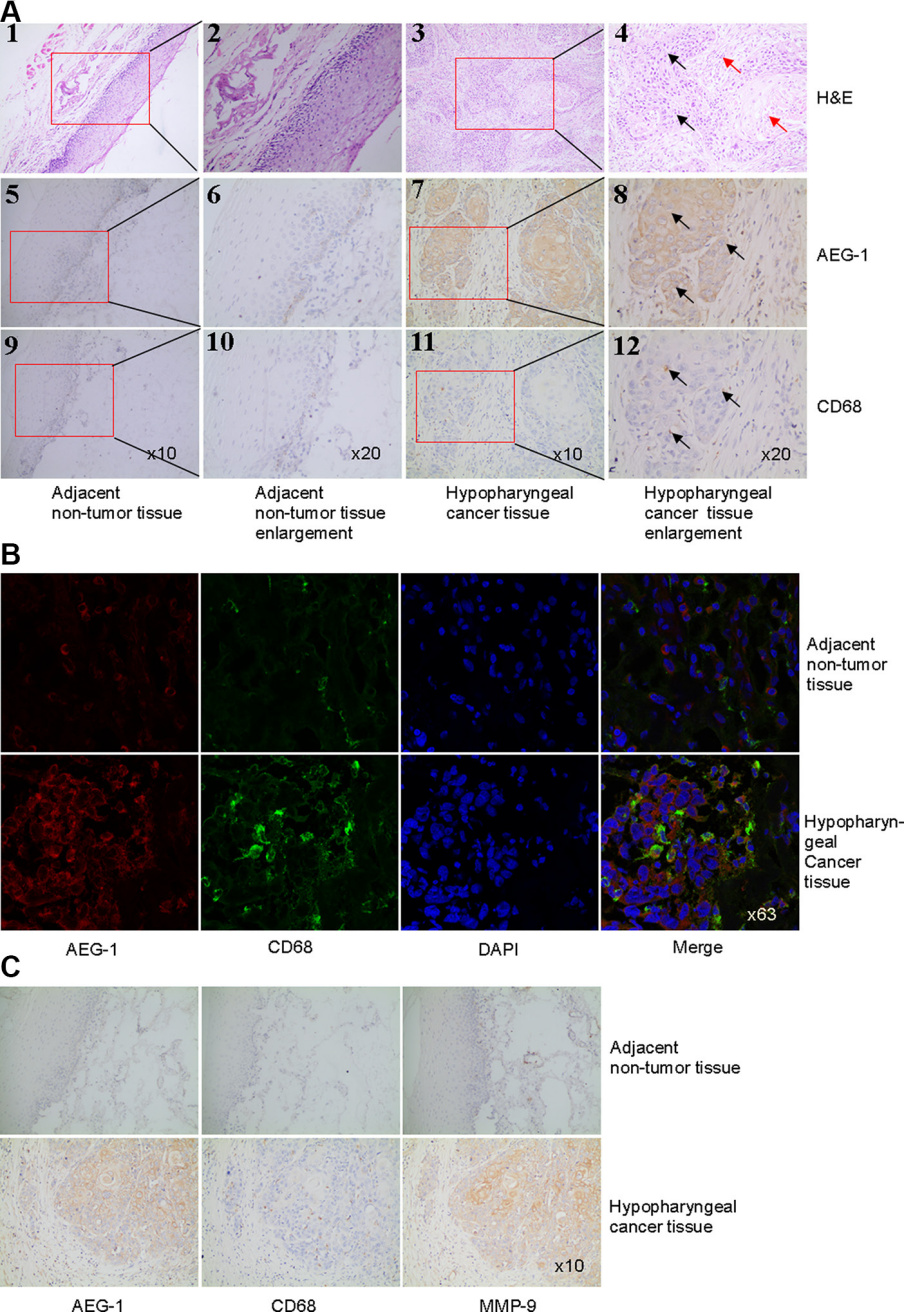
AEG-1 was highly expressed in TAM and its expression was associated with MMP-9 expression in hypopharyngeal cancer (**A**) H&E staining (1–4) and immunohistochemical staining of AEG-1 (brown staining, 5–8) and CD68 (brown staining, 9–12) were performed in hypopharyngeal cancer and non-tumor tissue. Higher magnifications of the boxed areas in (1), (3), (5), (7), (9), and (11) were shown in (2), (4), (6), (8), (10), and (12), respectively. The black arrows pointed out tumor nest and the red arrows pointed out tumor stroma in 4. The arrows in 8 and 12 indicated that AEG-1 was highly expressed in macrophages defined by CD68 in hypopharyngeal cancer. Magnification, × 10 and × 20. (**B**) Co-immunofluoresence staining with AEG-1 (red) and CD68 (green) in hypopharyngeal cancer and non-tumor tissue. Magnification, × 63. (**C**) Immunohistochemical staining of AEG-1, CD68, and MMP-9 in consecutive serial sections in non-tumor tissue and hypopharyngeal cancer. Magnification, × 10.

Histopathologic examination of hypopharyngeal cancer revealed a squamous cell carcinoma when hematoxylin and eosin (H&E) stain was used (Figure 1A1–1A4). We then examined the expression of AEG-1 in hypopharyngeal cancer samples by immunohistochemical staining. There was increased macrophage infiltration in tumor tissues compared with adjacent non-tumor tissues (Figure 1A5–1A12). In marked contrast to the adjacent non-tumor tissue, tumor samples from hypopharyngeal cancer showed higher expression of AEG-1 in macrophages defined by CD68 (Cluster of differentiation 68, a macrophage marker) staining (Figure 1A5–1A12). Next we performed co-immunofluorescence staining of AEG-1 and CD68 in sections of non-tumor tissue and tumor tissue samples. As shown in Figure 1B, AEG-1 was more expressed in macrophages of hypopharyngeal cancer samples than non-tumor samples. In order to investigate whether AEG-1 expression was associated with MMP-9 expression in hypopharyngeal cancer, we performed immunohistochemical staining of AEG-1, CD68 and MMP-9 (key regulator of tumor invasion) in serial sections. In contrast with adjacent non-tumor samples, both AEG-1 and MMP-9 were more highly expressed in hypopharyngeal cancer (Figure 1C). It was clearly indicated that AEG-1 expression markedly associated with MMP-9 expression in hypopharyngeal cancer (Figure 1C).

### THP-1 cells were induced to differentiate into macrophages

**Figure 2:**
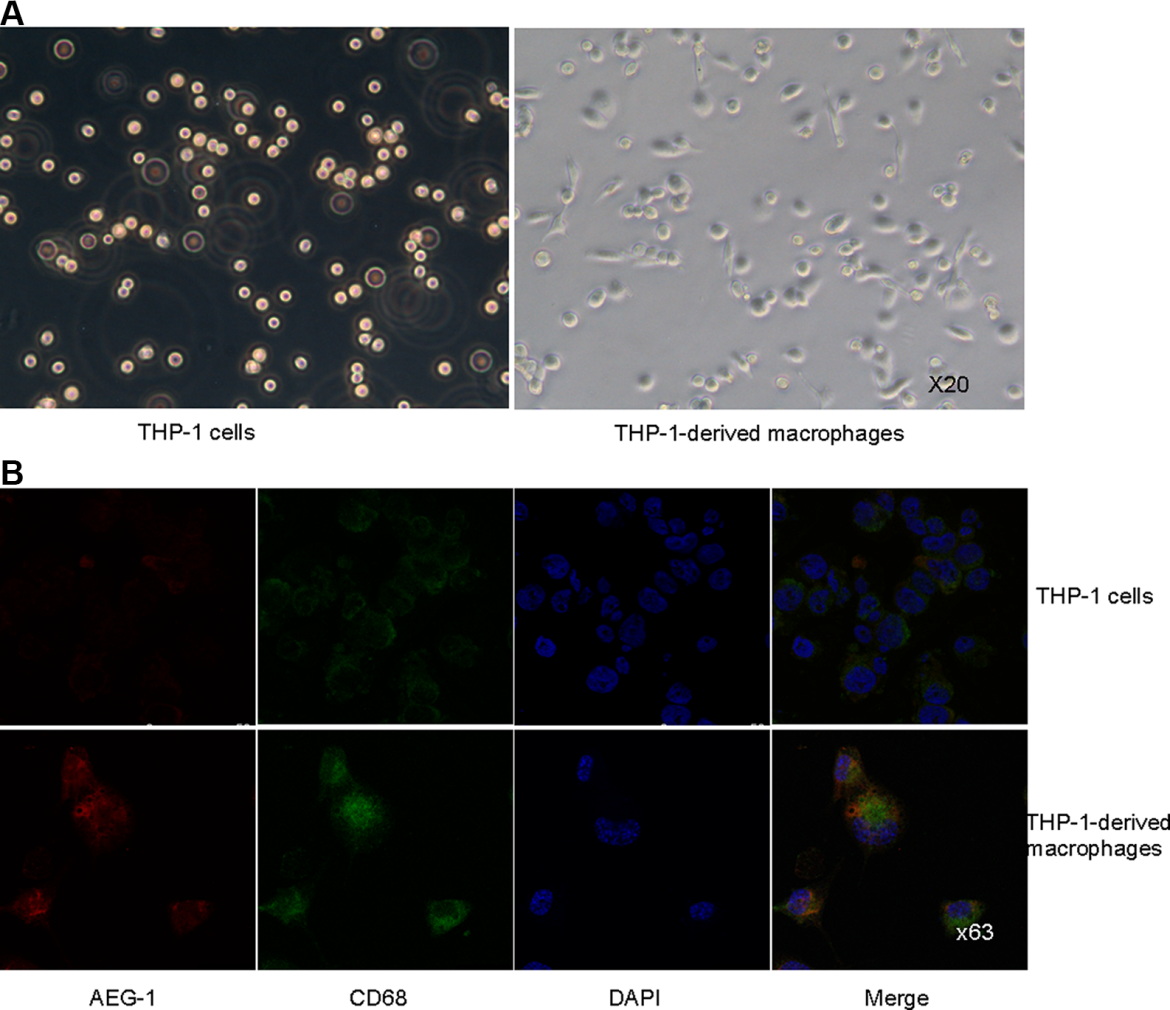
THP-1 cells were induced to differentiate into macrophages (**A**) Representative images of THP-1 cells untreated or treated with PMA. Magnification, × 20. (**B**) Co-immunofluorescence staining of AEG-1 (red) and CD68 (green) in THP-1 cells and PMA-stimulated macrophages. Magnification, × 63.

THP-1 cells, a human leukemia monocytic cell line, were treated with phorbol-12-myristate-13-acetate (PMA) at a concentration of 15 ng/ml. After 24 hour, over 90% PMA-stimulated cells adhered to culture dishes and morphological changes indicative of differentiation were observed (Figure 2A). Immunofluorescence analysis was performed using CD68 antibody to confirm the monocyte-to-macrophage differentiation of THP-1 cells. The expression of CD68 was clearly increased in PMA-stimulated macrophages than that in THP-1 cells (Figure 2B).

### AEG-1 expressed in THP-1-derived macrophages promoted the invasion of FaDu cells

**Figure 3:**
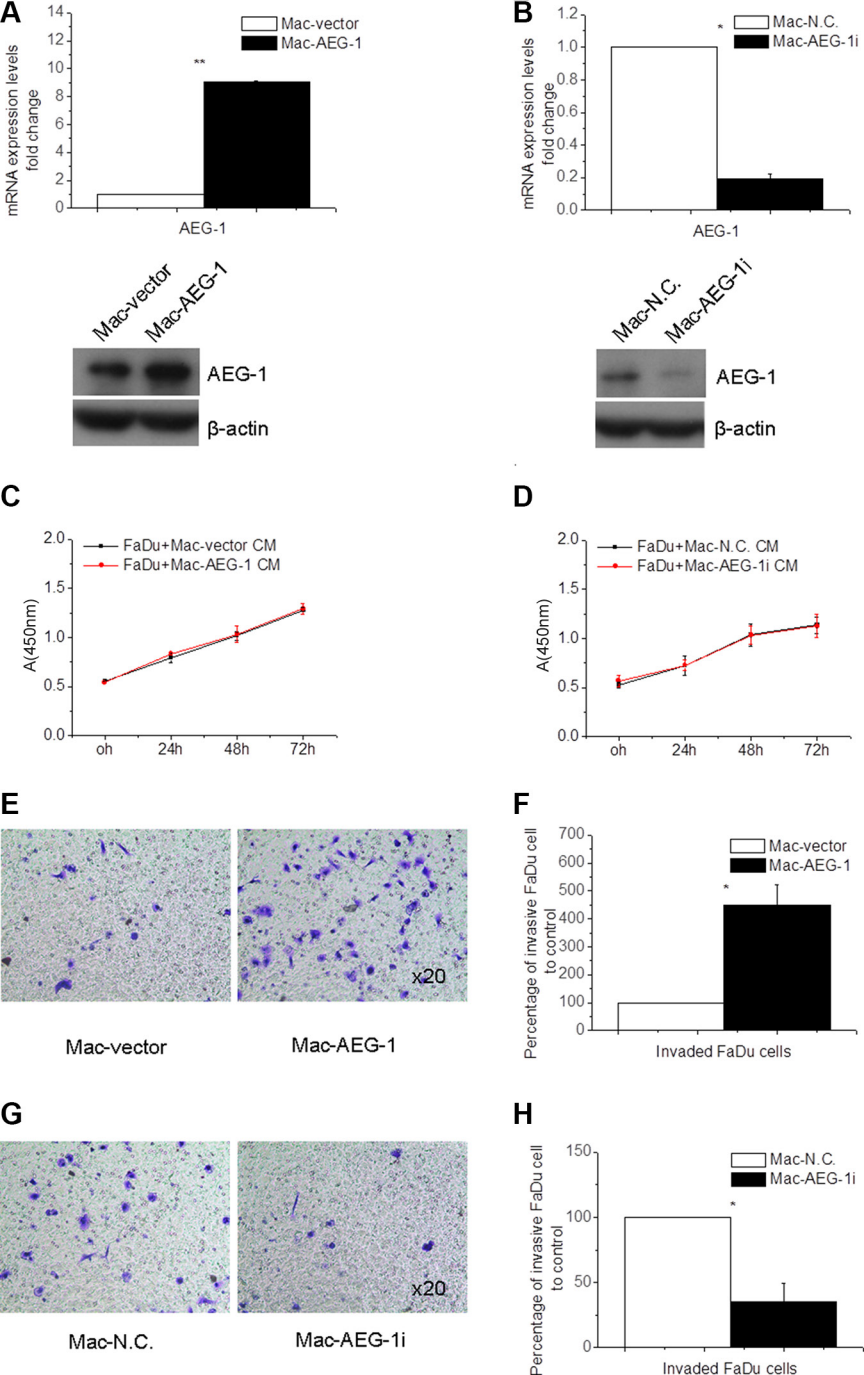
Macrophage AEG-1 promoted FaDu cell invasion (**A**) RT-QPCR and Western blot analysis of expression level of AEG-1 in Mac-AEG-1 and Mac-vector. (**B**) AEG-1 expression at mRNA and protein level in Mac-AEG-1i versus Mac-N.C. (**C**) The proliferation of Mac-AEG-1 CM- or Mac-vector CM-treated FaDu cells evaluated by CCK-8 assay. (**D**) The proliferation of Mac-AEG-1i CM- or Mac-N.C. CM-treated FaDu cells evaluated by CCK-8 assay. (**E**) Representative pictures of invaded FaDu cells when co-cultured with Mac-AEG-1 or Mac-vector respectively in invasion assay. Magnification, × 20. (**F**) Quantitative analysis of invaded FaDu cells when co-cultured with Mac-AEG-1 or Mac-vector respectively in invasion assay. (**G**) Representative pictures of invaded FaDu cells when co-cultured with Mac-AEG-1i or Mac-N.C. respectively in invasion assay. Magnification, × 20. (**H**) Quantitative analysis of invaded FaDu cells when co-cultured with Mac-AEG-1i or Mac-N.C. respectively in invasion assay. Data were presented as mean ± SEM. *, significantly different at *P* < 0.05; **, significantly different at *P* < 0.01.

In order to investigate the effect of AEG-1 expressed in macrophages on the invasion of FaDu cells, invasion assay was performed using macrophages transfected to overexpress or silence AEG-1. The expression of AEG-1 in THP-1-derived macrophages was detected by immunofluorescence assay (Figure 2B), real-time quantitative PCR (RT-QPCR), and Western blot analysis (Figure 3A–3B). Immunofluorescence assay showed that AEG-1 was located both in the cytoplasma and the nucleus (Figure 2B). Under basal condition, the expression of AEG-1 could be detectable in THP-1-derived macrophages. The levels of AEG-1 expression significantly increased at mRNA and protein level in macrophages transfected with pCMV-XL4 AEG-1 plasmid (Mac-AEG-1) compared with its control (Mac-vector) (Figure 3A). AEG-1 siRNA obviously suppressed the expression of AEG-1 in macrophages (Mac-AEG-1i) compared with macrophages transfected with control siRNA (negative control, N.C.) (Mac-N.C.) (Figure 3B).

First, CCK-8 assay was used to investigate the influence of AEG-1 expressed in macrophages on cell growth of FaDu cells. We collected condition media (CM) from Mac-AEG-1, Mac-vector, Mac-AEG-1i, and Mac-N.C. to treat FaDu cells for 24, 48, and 72 hours. Incubation of FaDu cells with Mac-AEG-1 CM for 24, 48, and 72 hours failed to increase the proliferation of FaDu cells compared with FaDu cells treated with Mac-vector CM (Figure 3C), and incubation with Mac-AEG-1i CM for these three time points also did not reduce the proliferation of FaDu cells (Figure 3D). Because of these results, cell invasion assay was performed with Mac-AEG-1 or Mac-AEG-1i for 24 hours to ignore the influence of cell viability.

We then conducted the invasion assays to observe the effect of AEG-1 expressed in macrophages on the invasion ability of FaDu cells. As shown in Figure 3E–3F, a significant increase in the number of invaded FaDu cells was observed when co-cultured with Mac-AEG-1 versus Mac-vector. Furthermore, we examined the effect of AEG-1 silence in macrophages on the invasion of FaDu cells. The invasion assay showed that knockdown of AEG-1 by siRNA in macrophages dramatically inhibited the invasion of FaDu cells compared with Mac-N.C. (Figure 3G–3H). The above results suggested that macrophage AEG-1 played an important role in promoting the invasion of FaDu cells.

### The effect of AEG-1 expression on macrophage polarization and MMP-9 expression in macrophages

**Figure 4:**
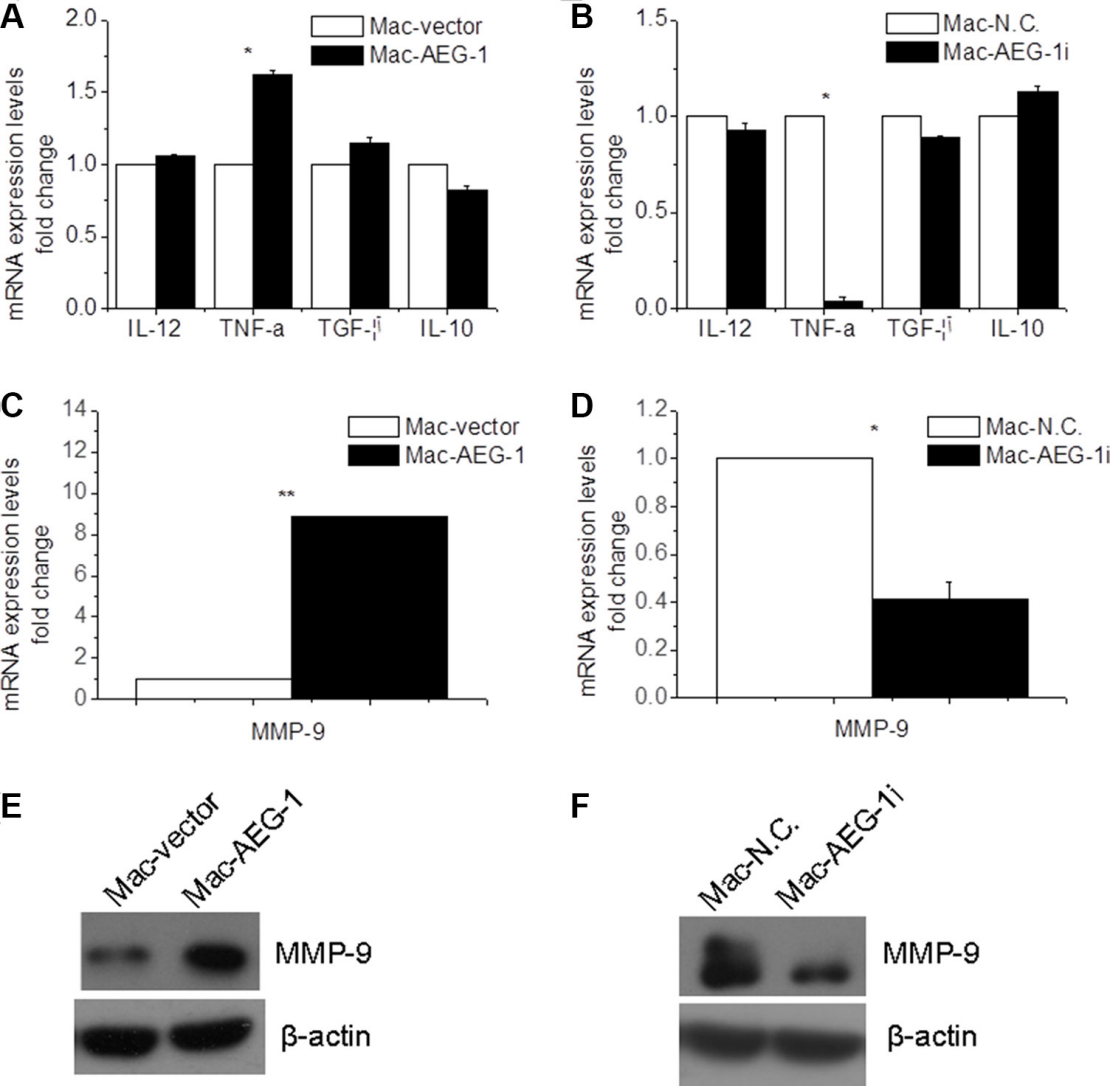
AEG-1 up-regulated MMP-9 expression in macrophages (**A**) Detection of IL-12, TNF-α, IL-10, and TGF-β expression in Mac-AEG-1 and Mac-vector. (**B**) Detection of IL-12, TNF-α, IL-10, and TGF-β expression in Mac-AEG-1i and Mac-vector. (**C**) MMP-9 mRNA expression in Mac-AEG-1 and Mac-vector. (**D**) MMP-9 mRNA expression in Mac-AEG-1i and Mac-N.C.. (**E**) Western blot analysis of MMP-9 protein expression in Mac-AEG-1 and Mac-vector. (**F**) Western blot analysis of MMP-9 protein expression in Mac-AEG-1i and Mac-N.C.. Data were presented as mean ± SEM. *significantly different at *P* < 0.05; **significantly different at *P* < 0.01.

To test whether the expression level of AEG-1 can affect macrophage polarization, RT-QPCR assay was conducted to determine the expression levels of Interleukin 12 (IL-12) and TNF-α, M1 (activated macrophages) markers and Interleukin 10 (IL-10) and transforming growth factor-β (TGF-β), M2 (alternatively activated macrophages) markers [19]. There was no significant difference in the expression level of IL-12, TGF-β, and IL-10 between Mac-AEG-1 and Mac-vector (Figure 4A). Moreover, the mRNA expression level of the above three molecules was not lower in Mac-AEG-1i than that in Mac-N.C. (Figure 4B). However, AEG-1 overexpression markedly increased the expression of TNF-α and AEG-1 silence led to a significant decrease in the expression of TNF-α in THP-1-derived macrophages (Figure 4A–4B).

It is well known that macrophages promote cancer cell invasion and metastasis, in part, through production of several factors such as MMP-9 which are responsible for matrix remodeling [20]. To understand the mechanism by which AEG-1 expressed in macrophages influenced FaDu cell invasion, we investigated the expression of MMP-9 in macrophages. MMP-9 mRNA expression was up-regulated in Mac-AEG-1 compared with Mac-vector (Figure 4C). AEG-1 siRNA significantly decreased MMP-9 mRNA expression in macrophages (Figure 4D). The identity of MMP-9 at protein level was further verified by Western blot analysis. We observed increased expression of MMP-9 in Mac-AEG-1 compared with Mac-vector (Figure 4E). As we expected, decreased MMP-9 expression was also found in Mac-AEG-1i (Figure 4F). Taken together, these data indicated that AEG-1 up-regulated MMP-9 expression in macrophages.

### Macrophage AEG-1-induced MMP-9 up-regulation and enhanced invasion ability in FaDu cell were mediated by STAT3 activation

**Figure 5:**
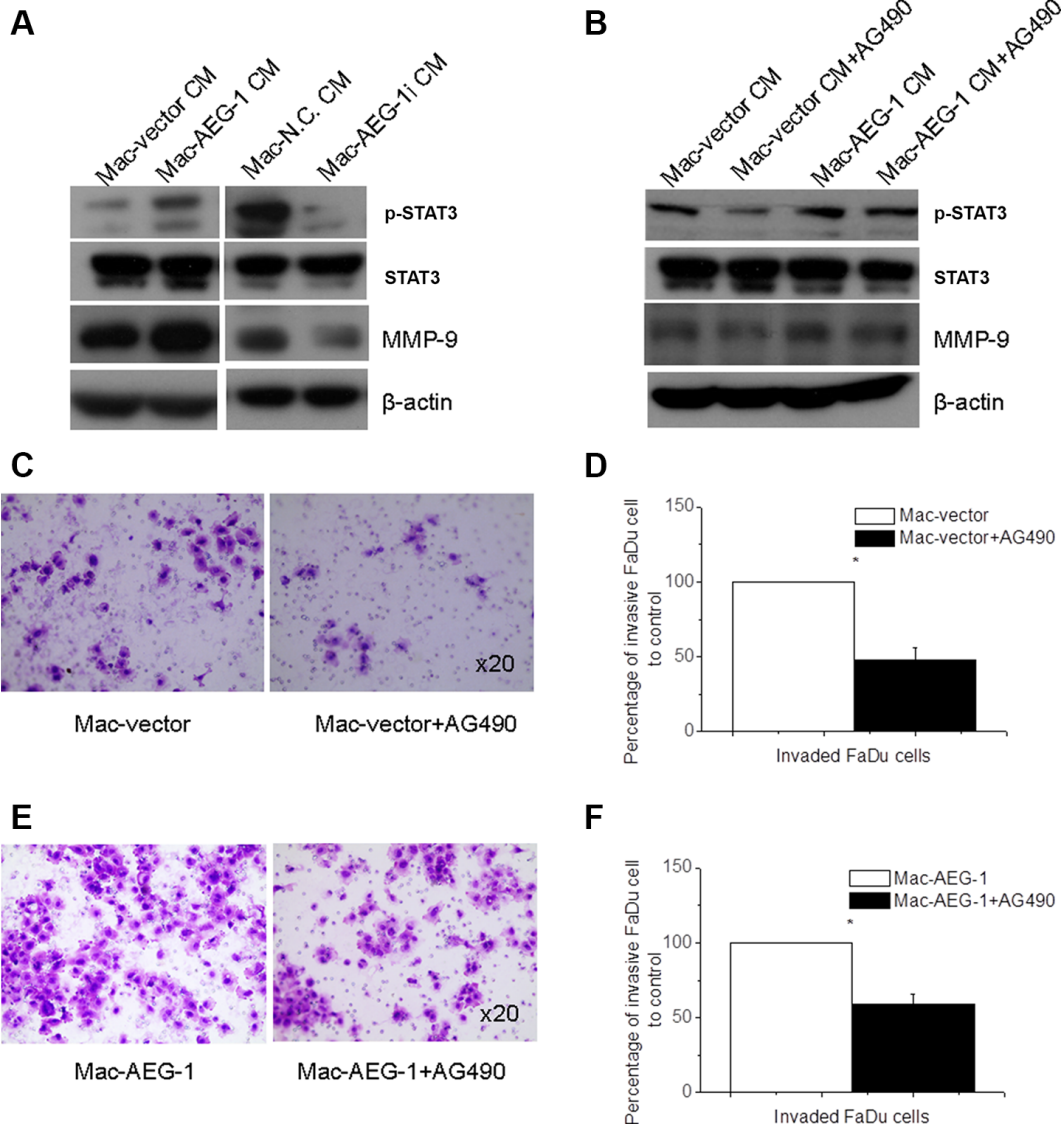
STAT3 activation in FaDu cells was responsible for macrophage AEG-1-induced increased MMP-9 expression and invasion ability of FaDu cells (**A**) Detection of STAT3 phosphorylation, STAT3, and MMP-9 expression in FaDu cells exposed to supernatants from Mac-vector, Mac-AEG-1, Mac-N.C., and Mac-AEG-1i. (**B**) Detection of STAT3 phosphorylation, STAT3, and MMP-9 expression in FaDu cells exposed to CMs of macrophages with/without AG490. (**C**) Representative pictures of invaded FaDu cells untreated or treated with AG490 when co-cultured with Mac-vector in invasion assay. Magnification, × 20. (**D**) Quantitative analysis of invaded FaDu cells untreated or treated with AG490 when co-cultured with Mac-vector in invasion assay. (**E**) Representative pictures of invaded FaDu cells untreated or treated with AG490 when co-cultured with Mac-AEG-1 in invasion assay. Magnification, × 20. (**F**) Quantitative analysis of invaded FaDu cells untreated or treated with AG490 when co-cultured with Mac-AEG-1 in invasion assay. Data were presented as mean ± SEM. *, significantly different at *P* < 0. 05.

According to the above results, AEG-1 induced MMP-9 expression in macrophages. However, it is not clear whether macrophage AEG-1 also affected the expression of MMP-9 in FaDu cells. Thus we collected CM from Mac-AEG-1, Mac-vector, Mac-AEG-1i, and Mac-N.C. and then used them to treat FaDu cells for 15 minutes. As displayed in Figure 5A, exposure to Mac-AEG-1 CM significantly increased the expression of MMP-9 in FaDu cells compared with FaDu cells treated with Mac-vector CM. Additionally, in supernatants of Mac-AEG-1i, MMP-9 expression was significantly suppressed in FaDu cells when compared with FaDu cells treated with Mac-N.C. CM.

We found that the pattern of STAT3 phosphorylation was similar to the pattern observed for MMP-9 in FaDu cells when they were treated with CM from Mac-AEG-1, Mac-AEG-1i, and their respective controls. Previous studies have demonstrated that STAT3 signaling mediated up-regulation of MMP-9 expression and conferred increased invasion ability in multi-drug-resistant breast cancer cells [21]. To examine the involvement of STAT3 activity in macrophage AEG-1-mediated MMP-9 up-regulation in tumor cells, we inhibited STAT3 activation in FaDu cells with AG490, which is a pharmacological inhibitor of the upstream STAT3 activator janus kinase. FaDu cells were treated for 24 hours with 20 μM/L AG490, and then stimulated with collected CM from Mac-AEG-1 and Mac-vector. Treatment with 20 μM/L AG490 markedly reduced the intensity of phospho-Tyr705-STAT3 (p-STAT3) and MMP-9 expression in FaDu cells treated with CM from either Mac-AEG-1 or Mac-vector (Figure 5B). However, compared with Mac-vector CM, Mac-AEG-1 CM alleviated AG490-induced suppression of MMP-9 expression (Figure 5B). These results suggested that STAT3 activation was involved in macrophage AEG-1-mediated up-regulation of MMP-9 expression in FaDu cells.

We further determined the functional role of activated STAT3 in macrophage AEG-1-induced invasion of FaDu cells. FaDu cells were treated with or without AG490 and then subjected to the invasion assay with Mac-AEG-1 or Mac-vector respectively. As shown in Figure 5C–5D, AG490 led to a decrease in the number of invaded FaDu cells when co-cultured with Mac-AEG-1 or Mac-vector. Moreover, Mac-AEG-1 alleviated the inhibitory effect of AG490 on FaDu cell invasion compared with Mac-vector (Figure 5E–5F). Taken together, STAT3 activation in FaDu cells was responsible for increased invasion ability of FaDu cells caused by macrophage AEG-1.

### NF-κB p65 was involved in macrophage AEG-1-induced IL-6 and MMP-9 expression in macrophages

**Figure 6:**
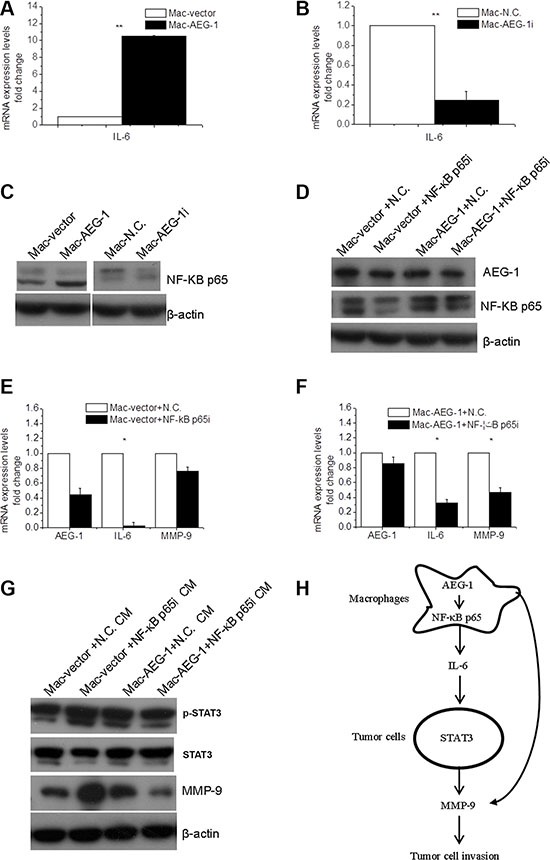
NF-κB p65 was involved in macrophage AEG-1-mediated up-regulation of IL-6 and MMP-9 in macrophages (**A**) Detection of IL-6 mRNA expression in Mac-AEG-1 and Mac-vector. (**B**) Detection of IL-6 mRNA expression in Mac-AEG-1i and Mac-N.C.. (**C**) The effect of AEG-1 on the expression of NF-κB p65 in macrophages. (**D**) The efficiency of simultaneous transfection of siRNA and plasmid was measured by Western blot. (**E**) Relative mRNA expression levels of AEG-1, IL-6, and MMP-9 in Mac-vector+N.C. and Mac-vector+NF-κB p65i. (**F**) Relative mRNA expression levels of AEG-1, IL-6, and MMP-9 in Mac-AEG-1+N.C. and Mac-AEG-1+NF-κB p65i. (**G**) Detection of p-STAT3, STAT3, and MMP-9 expression in FaDu cells exposed to supernatants from Mac-vector+N.C., Mac-vector+NF-κB p65i, Mac-AEG-1+N.C., and Mac-AEG-1+NF-κB p65i. (**H**) A schematic representation of macrophage AEG-1 promoting the invasion of FaDu cells. Data were presented as mean ± SEM. *, significantly different at *P* < 0.05; **, significantly different at *P* < 0.01.

Based on the above results, we inferred that macrophage AEG-1 may exert its effects by upregulating the expression of soluble factors such as IL-6, which was subsequently responsible for macrophage AEG-1-induced phosphorylation of STAT3 in FaDu cells (Figure 5A). When THP-1-derived macrophages infected with plasmids or siRNA were analyzed by RT-QPCR, we observed an increase in the expression of IL-6 mRNA in Mac-AEG-1 in comparison with Mac-vector (Figure 6A) and a decrease in IL-6 mRNA expression in Mac-AEG-1i compared with Mac-N.C. (Figure 6B).

It is generally believed that IL-6 produced in macrophages is one of the pro-inflammatory cytokines responsible for tumor progression, and is a key target gene of NF-κB p65 [22]. These lead us to wonder whether NF-κB p65 was involved in up-regulated expression of IL-6 by macrophage AEG-1. We first investigated the effect of AEG-1 expression on NF-κB p65 protein expression in macrophages. In contrast with Mac-vector, NF-κB p65 was markedly elevated in macrophages with enhanced AEG-1 expression (Figure 6C). Mac-AEG-1i showed a reduced expression of NF-κB p65 (Figure 6C). To demonstrate the role of NF-κB p65 in macrophage AEG-1-induced IL-6 expression, we then silenced NF-κB p65 expression with specific siRNA. Co-transfection of plasmid DNA with siRNA was performed and macrophages co-transfected with pCMV-XL4 vector plasmid and control siRNA, with pCMV-XL4 vector plasmid and NF-κB p65 siRNA, with pCMV-XL4-AEG-1 plasmid and control siRNA, and with pCMV-XL4-AEG-1 plasmid and NF-κB p65 siRNA were referred to be Mac-vector+N.C., Mac-vector+NF-κB p65i, Mac-AEG-1+N.C., and Mac-AEG-1+NF-κB p65i, respectively. The efficiency of co-transfection was confirmed by Western blot (Figure 6D). AEG-1 expression at mRNA level was examined in Mac-vector+N.C., Mac-vector+NF-κB p65i, Mac-AEG-1+N.C., and Mac-AEG-1+NF-κB p65i (Figure 6E–6F). Figure 6E displayed that the expression of NF-κB downstream gene IL-6 was much lower in Mac-vector+NF-κB p65i compared with that in Mac-vector+N.C.. However, in Mac-AEG-1+NF-κB p65i, the overexpression of IL-6 induced by AEG-1 was abrogated by NF-κB p65 siRNA (Figure 6F). The relative expression of IL-6 mRNA in Mac-AEG-1+NF-κB p65i was higher than that in Mac-vector+NF-κB p65i (Figure 6E–6F). These results demonstrated that NF-κB p65 was involved in the effect of AEG-1 on the regulation of IL-6 in macrophages. We further wondered whether NF-κB p65 was implicated in AEG-1-induced MMP-9 expression in macrophages. As shown in Figure 6E, MMP-9 expression in Mac-vector+NF-κB p65i siRNA was not significantly lower than that in Mac-vector+N.C.. But NF-κB p65 siRNA remarkably impaired the increase in MMP-9 expression induced by AEG-1 in macrophages (Figure 6F), which suggested that NF-κB p65 mediated AEG-1-induced MMP-9 expression in macrophages.

It has been demonstrated that macrophage AEG-1 mediated IL-6 expression through NF-κB p65 (Figure 6), we further collected CMs to investigate whether NF-κB p65 in macrophages affected macrophage AEG-1-induced elevated STAT3-MMP-9 signaling in FaDu cells. Western blot analysis showed that p-STAT3 and MMP-9 expression was definitely down-regulated in FaDu cells stimulated with Mac-AEG-1+NF-κB p65i compared with FaDu cells treated with Mac-AEG-1-N.C. (Figure 6G).

## DISCUSSION

So far, the majority of studies concerning the critical role of AEG-1 in tumor progression are investigated mainly in cancer cell types. This study indicated that AEG-1 was more highly expressed in TAM of hypopharyngeal cancer specimens compared with adjacent non-tumor tissues. Since high levels of the oncogene AEG-1 expression have been closely implicated in tumor invasion and metastasis [18], we hypothesized that AEG-1 expressed in macrophages may be involved in promoting tumor cell invasion. THP-1 cell line serves as a suitable *in vitro* cell model to study modulation of monocyte and macrophage function and used to investigate the effect of AEG-1 expressed in macrophages on tumor cell invasion. Here we elucidated that AEG-1 overexpressed in macrophages promoted the invasive capacity of FaDu cells via up-regulating MMP-9 expression in both macrophages and FaDu cells. The activation of STAT3 mediated macrophage AEG-1-induced the up-regulation of MMP-9 and enhanced invasion ability in FaDu cells. A schematic diagram of the underlying mechanism was displayed in Figure 6H.

Proteolytic enzymes, such as MMP-9, were required for invasion processes to degrade extracellular matrix, thereby facilitating the invasion of cancer cells to surrounding tissues [21, 23, 24]. Studies have found that MMP-9 is highly expressed in HNSCC [23, 25] and identified that MMP-9 expression is associated with lymph node metastasis [24] and poor outcome in laryngeal cancer [26]. In the present study, we demonstrated that elevated AEG-1 expression in hypopharyngeal cancer specimens was associated with increased expression of MMP-9. It is well-known that MMP-9 is a product of both tumor and stroma cells especially macrophages and plays a key role in cancer invasion, metastasis and angiogenesis. Our research revealed a novel mechanism that macrophage AEG-1 plays a major role in up-regulating the expression of MMP-9 in both macrophages and FaDu cells.

During malignant transformation, STAT3 is constitutively activated in about 70% of solid and hematological tumors by tyrosine phosphorylation [27]. In various cancers, overexpression of p-STAT3 correlated with increased invasion and metastasis [20]. Evolving evidences implicate that STAT3 activation is involved in MMP-9 expression and matrix remodeling [28, 29] and thus confers enhanced invasion ability in drug-resistant cancer cells [21]. This study showed that activation of STAT3 in FaDu cells partially mediated macrophage AEG-1-driven up-regulation of MMP-9 in FaDu cells. This is the first to demonstrate that STAT3-MMP-9 pathway was partially responsible for macrophage AEG-1-induced cancer cell invasion.

Based on numerous studies, TAM are thought to be associated with increased angiogenesis and metastasis through expression of a variety of cytokines and growth factors [20]. NF-κB is an important regulator of gene transcription in macrophages and NF-κB activation in macrophages has been shown to contribute to carcinogenesis in several models of inflammation-associated cancer [30, 31]. Gene chip analysis reveals that AEG-1 overexpression leads to up-regulation of several NF-κB downstream genes, including IL-6 [9]. Consistent with the findings of previous research [9], another study demonstrates that AEG-1 knockdown could lead to the inactivation of NF-κB and down-regulation of MMP-9 [32]. Consistent with previous study [14], we showed that AEG-1 was required for NF-κB p65 activation in macrophages. Our study emphasized the importance of NF-κB p65 in AEG-1-induced up-regulation of IL-6 and MMP-9 in macrophages. Direct co-culture of tumor cells and macrophages shows that various factors secreted by activated macrophages, including IL-6, activate STAT3 in tumor cells [20]. In other words, STAT3 activation in tumor cells mediates the effect of macrophages on tumor cells [33]. In this study, it is observed that macrophage AEG-1 up-regulated the expression of IL-6, which may be responsible for STAT3 activation in tumor cells. However, the contribution of macrophage AEG-1 to tumor progression was associated with many other cytokines and growth factors, more researches are required to be done to investigate the underlying mechanism that how AEG-1 in macrophages exerts its full effect on the production of IL-6 and other cytokines as well as growth factors closely involved in tumor cell invasion and metastasis.

In 2000, Mills et al. proposed that macrophages consisted of two classes: M1-macrophages and M2-macrophages. In some tumors investigated, TAM are similar to M2 macrophages [34], which are characterized as IL-12^low^IL10^high^. However, this binary definition is limiting and unavailable in the complex tumor microenvironment [35]. Furthermore, recent gene profiling experiments support that macrophages could be either M1 or M2 in tumor microenvironment [36, 37]. In this study, AEG-1 overexpression or silence resulted in no change in the expression of both M1 and M2 markers except TNF-α. It has been demonstrated that TNF-α, produced primarily by macrophages, is required for the early stages of carcinogenesis [38]. Besides TNF-α, another inflammation factor, IL-6, whose expression was elevated in macrophages with AEG-1 overexpression, was also reported contributing to the creation of an inflammatory environment [35] and resulting in a chronic colitis and invasive colonic adenocarcinomas [39]. Thus, macrophage polarization was not affected by altered expression of AEG-1 in macrophages. But AEG-1 overexpression did induce macrophages to produce crucial inflammatory factors such as IL-6 and TNF-α who are important in promoting tumor progression, invasion and metastasis [39, 40].

Taken together, elevated expression of AEG-1 was found in TAM of hypopharyngeal cancer. It is demonstrated that macrophage AEG-1 promoted FaDu cell invasion through up-regulation of MMP-9 and IL-6 in macrophages via NF-κB p65. The supernatants from Mac-AEG-1 stimulated STAT3 activation which was responsible for up-regulation of MMP-9 and enhanced invasion capability in FaDu cells. Our study unraveled the important role of AEG-1 expressed in macrophages on hypopharyngeal cancer cell invasion and could potentially represent a potentially therapeutic target.

## MATERIALS AND METHODS

### Histological analysis

Formalin-fixed, paraffin-embedded hypopharyngeal cancer tissue blocks were obtained from the archive of the Department of otorhinolaryngology head and neck surgery, in accordance with ethical approval and institutional guidelines of the Shandong Provincial Hospital affiliated to Shandong University. Sections of each tissue sample were stained with H&E. Diagnosis of squamous cell carcinoma was based on pathological and/or cytological evidence. Tissue sections, which included both tumor and adjacent non-tumor parts, were cut to 3μm in thickness. Consecutive sections were deparaffinized and rehydrated, and antigen retrieval was done by incubating the slides at 95°C for 20 minutes in Target Retrieval Solution (ZSJB-BIO) according to the manufacturer's instructions. Adjacent serial sections were blocked for 10 minutes at room temperature to block non-specific binding and then incubated with rabbit anti-human AEG-1 antibody (Abcam), mouse anti-human CD68 antibody (ZSJB-BIO) or mouse anti-MMP-9 antibody (ZSJB-BIO) at 4°C overnight. HRP-conjugated polyclonal goat anti-rabbit and goat anti-mouse antibodies were used as secondary antibodies. HRP activity was detected with 3,3 0-diaminobenzidine (DAB) substrate chromogen (ZSJB-BIO). All histopathological images were taken with an Olympus BX53 microscope.

### Cell culture and differentiation

The human monocyte line THP-1 and FaDu cell lines were both obtained from the Cell Bank, Chinese Academy of Sciences. FaDu cells were cultured in DMEM/F12 supplemented with 10% Fetal bovine serum (FBS) and incubated at 37°C with 5% CO2. THP-1 cells were cultured in RPMI1640 containing 10% FBS and maintained at a concentration of 2∼10 × 10^5^ cells/ml. As described previously [41], THP-1 cells at a density of 4∼6 × 10^5^ cells/ml were treated with 15 ng/ml PMA (Sigma-Aldrich) diluted in dimethyl sulfoxide for 24 hours to differentiate into macrophages. Cells were then maintained in complete medium and left to recover for several hours.

### Cell transfection with plasmids and siRNAs

The pCMV-XL4 AEG-1 plasmid was purchased from OriGene and specific siRNA targeting AEG-1 or NF-κB p65 was synthesized from GeneChem Co., Ltd. According to the standard procedure of X-tremeGENE HP DNA Transfection Reagent (Roche), pCMV-XL4 AEG-1 plasmid or empty vector plasmid was transfected into THP-1-derived macrophages. Macrophages were transfected with siRNA using X-tremeGENE siRNA Transfection Reagent (Roche), which was also available for co-transfection of siRNA and plasmid DNA into macrophages.

### Immunofluorescence

THP-1 cells in suspension were collected, centrifuged and resuspended in 100 μl fresh medium. These cells and tissue samples were embedded in Tissue-Tek OCT compound (Sakura Seiki) and cryosectioned. THP-1 cells and THP-1-derived macrophages were fixed in 4% paraformaldehyde. Tissue specimens and macrophages were permeabilized with 0.1% Triton X-100 in PBS for 15 minutes. They were blocked at room temperature for 1 hour and then incubated with primary antibodies overnight at 4°C. Co-immunofluorescence staining was done with anti-AEG-1 (Abcam) and anti-CD68 (ZSJB-BIO) antibodies as previously described [42]. Sections were rinsed in PBS and incubated with either Alexa Fluor 546-conjugated goat anti-rabbit (Invitrogen) or DyLight-488 conjugated goat anti-mouse secondary antibodies (ZSJB-BIO) for 1 hour at room temperature. Nuclei were counterstained with 4′,6-diamidino-2-phenylindole (DAPI). The images were then captured by confocal microscope (TCS SPE; Leica, Germany).

### CCK-8 assay

Cell viability was detected with CCk-8 assay. FaDu cells were seeded in 96-well culture plates. The plates were placed for 24 hours in the incubator and the culture medium was changed to collected CM. CCK-8 assays were performed 24, 48, and 72 hours after CM treatment. At the time of the CCK-8 assay, added 10 μL CCK-8 solution to each well of the plate and incubated the plate for 2 hours at 37°C. Absorbance was measured at 450 nm using a microplate reader (BioTek). The results were used to measure cell growth.

### Invasion assay

THP-1 cells were seeded in 24-well plates and induced to differentiate into macrophages, which was followed by transfection. For the invasion assay, the inserts of modified Boyden Chambers (BD Bioscience) were pre-coated with 50 μL Matrigel (BD Bioscience, 1/3 diluted in DMEM/F12) for 30 minutes at 37°C. Prepare cell suspension in serum-free DMEM/F12 and 200 μl cell suspensions containing 2.5 × 10^4^ FaDu cells were immediately added into the upper inserts of Boyden Chambers. Twenty-four hours after co-culture, invaded cells were fixed with 4% formaldehyde and stained with crystal violet for 15 minutes. Invasion was determined by counting of cells that migrated onto the lower side of the membranes using a microscope. The experiments were performed in triplicate and five random microscopic fields per well were counted.

### Isolation of total RNA and RT-QPCR

Total RNA was prepared with the TRIzol reagent (Invitrogen) and reverse transcription (Takara) was performed following the manufacturer's protocol (Takara). Using forward and reverse primers, RT-QPCR assay was performed in the presence of GoTaq^®^ qPCR Master Mix (Promega) in accordance with the manufacturer's instructions. The relative quantitation of gene mRNA expression was normalized to β-actin in the same sample. The sequences of the primers were as follows:

Human β-actin: forward 5′-CCAACCGCGAGAAG ATGA-3′, reverse 5′- CCAGAGGCGTACAGGGATAG-3′; Human AEG-1: forward 5′-AACAGCAAAGCAGCCAC CAGAG-3′, reverse 5′-CAGGAAGGAAGGCTGGAA GAGTG-3′; Human MMP-9: forward 5′- TTGACAGCG ACAAGAAGTGG-3′, reverse 5′-GCCATTCACGTCGT CCTTAT-3′; Human IL-6: forward 5′-AAGCCAGAGCTG TGCAGATGAGTA-3′, reverse 5′-TGTCCTGCAGCCAC TGGTTC-3′; Human IL-12: forward 5′-ACCCTGACCA TCCAAGTCAAA-3′, reverse 5′-TTGGCCTCGCATC TTAGAAAG-3′; Human TNF-α: forward 5′-CCCAGGG ACCTCTCTCTAATC-3′, reverse 5′-GCTGGTTATCTCT CAGCTCCA-3′; Human TGF-β: forward 5′-CAACA ATTCCTGGCGATACC-3′, reverse 5′-GAACCCGTTGA TGTCCACTT-3′; Human IL-10: forward 5′-GACTTTA AGGGTTACCTGGGTTG-3′, reverse 5′-TCACATG CGCCTTGATGTCTG-3′.

### Western blot

Cells were lysed in a RIPA buffer to obtain total proteins. Protein lysates were separated by SDS-PAGE and transferred to PVDF membranes. After blocking with 5% non-fat milk, membranes were incubated with primary antibodies overnight at 4°C and then with linked secondary antibodies. Primary antibodies were listed as followed: AEG-1 (Abcam), MMP-9 (Abcam), P-STAT3 (Tyr705) (Cell Signaling), STAT3 (BD Bioscience), NF-κB p65 (Santa Cruz), β-Actin (ZSJB-BIO).

### Statistical analysis

All of the experiments were done at least three times. The results were depicted as the mean ± SEM (standard error of the mean). All statistical analyses were performed using Student's unpaired and paired *t* tests.
